# COVID-19 Vaccine Fact-Checking Posts on Facebook: Observational Study

**DOI:** 10.2196/38423

**Published:** 2022-06-21

**Authors:** Haoning Xue, Xuanjun Gong, Hannah Stevens

**Affiliations:** 1 Department of Communication University of California, Davis Davis, CA United States

**Keywords:** COVID-19 vaccine, fact checking, misinformation correction, sentiment analysis, social media, COVID-19, vaccination, misinformation, health information, online information, infodemic, public sentiment

## Abstract

**Background:**

Effective interventions aimed at correcting COVID-19 vaccine misinformation, known as fact-checking messages, are needed to combat the mounting antivaccine infodemic and alleviate vaccine hesitancy.

**Objective:**

This work investigates (1) the changes in the public's attitude toward COVID-19 vaccines over time, (2) the effectiveness of COVID-19 vaccine fact-checking information on social media engagement and attitude change, and (3) the emotional and linguistic features of the COVID-19 vaccine fact-checking information ecosystem.

**Methods:**

We collected a data set of 12,553 COVID-19 vaccine fact-checking Facebook posts and their associated comments (N=122,362) from January 2020 to March 2022 and conducted a series of natural language processing and statistical analyses to investigate trends in public attitude toward the vaccine in COVID-19 vaccine fact-checking posts and comments, and emotional and linguistic features of the COVID-19 fact-checking information ecosystem.

**Results:**

The percentage of fact-checking posts relative to all COVID-19 vaccine posts peaked in May 2020 and then steadily decreased as the pandemic progressed (*r*=–0.92, *df*=21, *t*=–10.94, 95% CI –0.97 to –0.82, *P*<.001). The salience of COVID-19 vaccine entities was significantly lower in comments (mean 0.03, SD 0.03, *t*=39.28, *P*<.001) than in posts (mean 0.09, SD 0.11). Third-party fact checkers have been playing a more important role in more fact-checking over time (*r*=0.63, *df*=25, *t*=4.06, 95% CI 0.33-0.82, *P*<.001). COVID-19 vaccine fact-checking posts continued to be more analytical (*r*=0.81, *df*=25, *t*=6.88, 95% CI 0.62-0.91, *P*<.001) and more confident (*r*=0.59, *df*=25, *t*=3.68, 95% CI 0.27-0.79, *P*=.001) over time. Although comments did not exhibit a significant increase in confidence over time, tentativeness in comments significantly decreased (*r*=–0.62, *df*=25, *t*=–3.94, 95% CI –0.81 to –0.31, *P*=.001). In addition, although hospitals receive less engagement than other information sources, the comments expressed more positive attitudinal valence in comments compared to other information sources (b=0.06, 95% CI 0.00-0.12, *t*=2.03, *P*=.04).

**Conclusions:**

The percentage of fact-checking posts relative to all posts about the vaccine steadily decreased after May 2020. As the pandemic progressed, third-party fact checkers played a larger role in posting fact-checking COVID-19 vaccine posts. COVID-19 vaccine fact-checking posts continued to be more analytical and more confident over time, reflecting increased confidence in posts. Similarly, tentativeness in comments decreased; this likewise suggests that public uncertainty diminished over time. COVID-19 fact-checking vaccine posts from hospitals yielded more positive attitudes toward vaccination than other information sources. At the same time, hospitals received less engagement than other information sources. This suggests that hospitals should invest more in generating engaging public health campaigns on social media.

## Introduction

### Background

As of May 4, 2022, the novel COVID-19 outbreak had caused 994,551 deaths and 81,574,159 cases in the United States [1]. Compared to COVID-19 deaths per capita in developed countries with similarly aged populations (ie, the United Kingdom, France, Spain, Canada), the United States has the highest number of deaths per 100,000 people [1]. This may be because, despite widespread vaccine availability, the United States has the lowest rate of individuals who are fully vaccinated and boosted (30%) compared to developed countries with similarly aged populations (eg, 52% in Canada). Indeed, those who haven’t received all 3 doses of the vaccine account for the majority of deaths and severe cases in the United States [2-4]. Furthermore, vaccine hesitancy has constrained public health officials’ efforts to mitigate the pandemic through herd immunity [5,6].

Thus, effectively communicating the necessity of getting the COVID-19 vaccine is essential to mitigating the COVID-19 pandemic. Although some public officials have endorsed the COVID-19 vaccine, others have fostered vaccine hesitancy by broadcasting misinformation (ie, inaccurate health information), which is often disseminated widely via social media [7,8]. The prevalence of US adults who indicate they primarily get news information from social media (ie, 68%) [9] has given rise to an *infodemic*, wherein public confidence in the COVID-19 vaccine is shaken by the overload of COVID-19 misinformation on social media [10-12]. Indeed, Loomba et al found that exposure to COVID-19 vaccine misinformation significantly decreases the intention to receive the COVID-19 vaccine [13]. Thus, effective interventions aimed at correcting COVID-19 vaccine misinformation, known as fact-checking messages, are needed to combat the mounting antivaccine infodemic and alleviate vaccine hesitancy [14,15].

Research efforts have therefore focused on experimentally testing the efficacy of COVID-19 vaccine misinformation fact-checking messages, finding that accurate misinformation correction messages can effectively mitigate health misinformation in certain contexts, namely when the message is from a credible information source (ie, health institutions, research institutions, and news media) [16-19]. Thus, although we know fact checking from credible sources can be effective, the extent to which credible information sources share fact-checking information and the ways in which the public engages with COVID-19 vaccine fact checks in naturalistic social media environments remain neglected in the literature. This work aims to fill this gap by investigating (1) the changes in the public's attitude toward COVID-19 vaccines over time, (2) the effectiveness of COVID-19 vaccine fact-checking information on social media engagement and attitude change, and (3) the emotional and linguistic features of the COVID-19 vaccine fact-checking information ecosystem. This study expands our knowledge of the COVID-19 vaccine information environment on social media and contributes to our understanding of the effectiveness of COVID-19 vaccine fact-checking messages on the public's attitudes toward the COVID-19 vaccine. A novel contribution of this study lies in the usage of entity-targeted sentiment powered by Google Cloud Natural Language AI (where AI is artificial intelligence) that enables us to capture the exact attitude toward the COVID-19 vaccine among the public [20].

### The Public's Attitude Toward COVID-19 Vaccines

Vaccine attitude determines people’s intention to vaccinate and the consequential vaccine uptake behaviors [21,22]. However, exposure to misinformation can cause a decline in people’s vaccination intention to receive COVID-19 vaccines in both the United Kingdom and the United States [13]. Thus, to assess how effective vaccination campaigns are against COVID-19 misinformation, such as fact-checking messages on social media, it is important to investigate the actual impact on the public's attitude toward the COVID-19 vaccine. Previous experimental studies have shown that fact-checking interventions can promote people’s positive attitude toward vaccines and increase the accuracy of beliefs about vaccination [12,23]. However, empirical observational evidence for fact-checking messages’ effects on the public's attitude toward the COVID-19 vaccine in the real world is still lacking. In this study, we explore this question by examining how the public's attitude toward the COVID-19 vaccine, as reflected by attitudinal linguistic markers related to COVID-19 vaccine–related entities in comments attached to fact-checking posts, changes as a response to fact-checking posts over time. Accordingly, we ask research question (RQ)1a: *How does the attitude toward the COVID-19 vaccine in fact-checking comments change over time?*

In addition, it is also important to investigate the attitude toward COVID-19 vaccine fact-checking messages itself. Emotionally charged messages are found to influence vaccination intent more than facts and statistics [24], and discrete emotions impact vaccine behaviors differently [25]. A 2020 study found that emotionally positive COVID-19 health messages predict compliance with COVID-19 public health guidelines when the messages evoke highly positive responses [26]. In contrast, desensitization to emotionally charged, negatively valenced COVID-19 messages can prompt folks to become disengaged, unmotivated to take protective action, and susceptible to health misinformation [27-29]. Thus, the extent to which credible health sources broadcast positively valenced, emotionally charged messages is consequential; accordingly, we pose the following RQ1b: *How does the attitude toward the COVID-19 vaccine in fact-checking posts change over time?*

### Effects of Fact-Checking Information Sources

We know politicians are a prevalent source of vaccine misinformation on social media (Featherstone et al. [17-19]), and the credibility of the source of vaccine information can determine fact-checking message efficacy. Indeed, health information from sources with authority (eg, health institutions) are perceived as more credible [30]. However, the extent to which credible information sources share misinformation corrections and the ways in which the public engages with COVID-19 vaccine fact-checking messages in naturalistic social media environments remain unclear. Thus, we pose the following RQ2: *How do different information sources of COVID-19 vaccine fact-checking posts influence (1) the public's attitude toward the COVID-19 vaccine and (2) social media engagement with fact-checking posts?*

### Emotional Trends and Linguistic Features in COVID-19 Fact-Checking Posts

In addition to the public's valenced attitude toward the COVID-19 vaccine, varied discrete emotions may reveal more about the specific attitudes or concerns. Different discrete emotions have different effects on the vaccine-hesitant. For example, vaccine-hesitant users are more likely than provaccine users to express anger in posts and replies [31]. For health promotion information, heightened anxiety in protective health messages can encourage the hesitant to vaccinate [32]. Thus, we are interested in the specific discrete emotions that emerge in posts and comments over time. Thus, we posit RQ3: *Which discrete emotions manifest in COVID-19 vaccine fact-checking posts and comments over time?*

Although staunch antivaxxers exist, a prominent group of Americans who understand vaccine importance are hesitant to take it because of uncertainty about the safety of the vaccine’s rushed development [33]. Thus, correcting misinformation with confident messages (ie, low tentativeness, high certainty) is an essential component of restoring public trust in the vaccine’s safety and efficacy. Furthermore, health threat messages with logical, actionable steps for alleviating the health threat can encourage protective behaviors, such as encouraging vaccination [32]. Yet, the extent to which credible sources of health information projected confidence and logic in messages throughout the pandemic is unclear, particularly at the beginning of the vaccine rollout, when even health experts were uncertain about aspects of the vaccine (eg, how the vaccine works in those with COVID-19 mortality risk factors, which vaccine is most effective) [34]. In addition, how such subtle linguistic features are present in average social media users' remarks informs us how the public's attitude toward the COVID-19 vaccines has evolved over time. As such, we raise RQ4: *How do linguistic features of confidence, tentativeness, and analytical thinking in the COVID-19 vaccine fact-checking posts and comments vary over time?*

## Methods

### Data Set

To fill these gaps in the literature, we collected a data set of 12,553 COVID-19 vaccine fact-checking posts and their associated comments (N=122,362) from January 2020 to March 2022. Facebook was selected because it is 1 of the most popular social media platforms worldwide with a significant presence of both misinformation and fact-checking information [35].

### Collecting Facebook Posts Using CrowdTangle

We leveraged Meta’s *CrowdTangle* tool to (1) identify relevant sources of COVID-19 vaccine information and (2) collect Facebook posts related to the COVID-19 vaccine created between January 1, 2020, and March 10, 2022 [36]. CrowdTangle is a data-tracking platform owned by Meta (Facebook's parent company) that monitors social media public conversations and related data.

**Figure 1:**
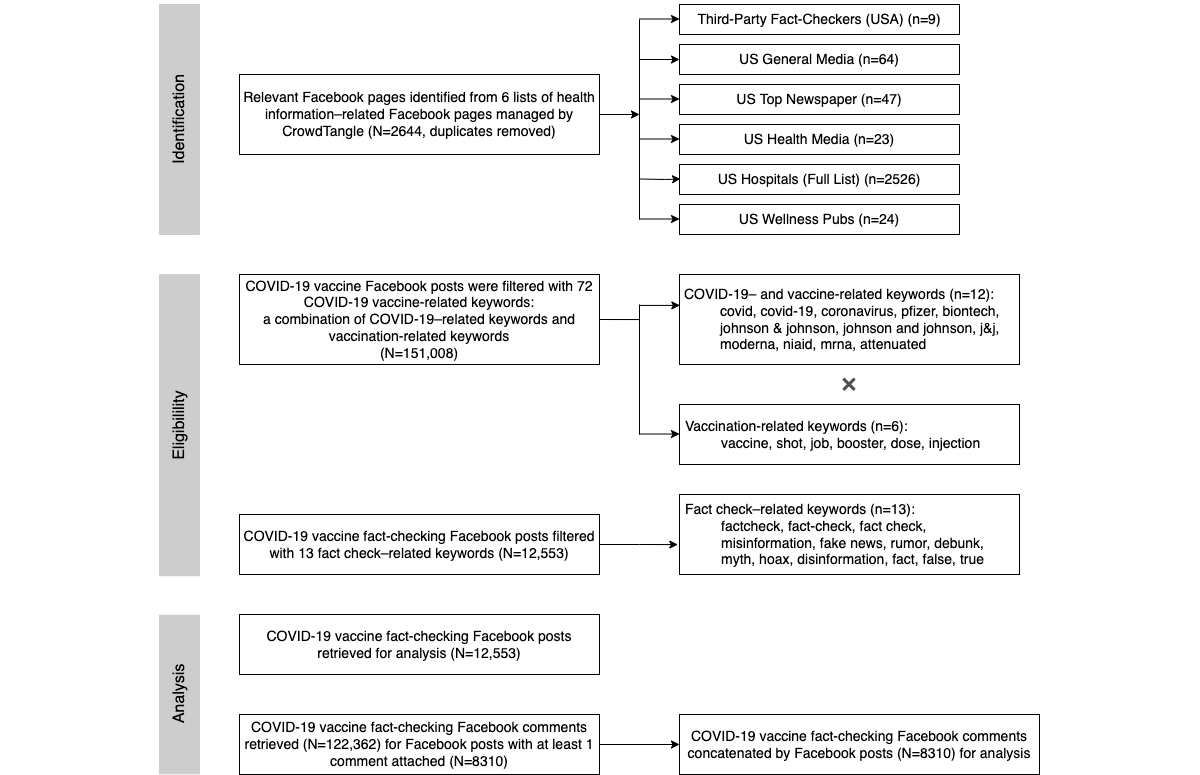
Process to identify relevant lists of Facebook pages, collect Facebook posts and comments, and filter relevant Facebook posts.

CrowdTangle tags public Facebook pages based on several attributes, including the primary language of the content, the country the content is geared toward, and the type of entity that owns the page (eg, health influencer, top newspaper). We curated a list of pages belonging to categories related to health information sharing, namely third-party fact checkers, general media sources, top newspaper sources, health influencers, health media, hospitals, and wellness publications (Figure 1). To keep the framework parsimonious, the 6 categories were further aggregated into 4 category types: (1) US news media (including US general media and the top newspaper), (2) third-party fact checkers, (3) US health media (including US health media and wellness publications), and (4) US hospitals. Only English Facebook pages geared toward US audiences were retained. In total, there were 2644 unique pages obtained from the categories, with duplicates removed (n=49).

Second, we mined the total number of COVID-19 vaccine–related posts (N=151,008) and the accompanying post metadata provided with Crowdtangle (ie, date, number of shares, comments, and emoticon reactions) from the curated list of health-related Facebook pages (N=2644) [37]. Posts were retained if they contained both (1) at least 1 COVID-19– or vaccine-related keyword (eg, “mRNA,” “coronavirus”) and (2) at least 1 vaccine-related keyword (eg, “vaccines,” “booster,” “dose”) in either the body of the post or a Uniform Resource Locator (URL) shared in the post. See Table S1 in Multimedia Appendix 1 for an overview.

Further, we extrapolated posts containing at least 1 (8%) of 13 fact-checking keywords (eg, “debunk,” “hoax”) to distill fact-checking posts (N=12,553) from the larger data set (Multimedia Appendix 1, Table S2). COVID-19 vaccine fact-checking posts (N=12,553) from 1226 different Facebook pages were retained for further analysis.

### Collecting Facebook Comments Using Facepager

Although CrowdTangle makes some metadata available (ie, post date, number of shares, and number of Facebook emoticon reactions), it does not provide access to comment data. We used an automatic data collection software called *Facepager* to retrieve up to 25 of the highest-ranked comments (ie, “top 25 comments”) attached to each Facebook post in the data set [37,38]. We retrieved 122,362 comments associated with 12,553 COVID-19 vaccine fact-checking posts. Comments attached to a post were concatenated as a single textual observation; not all posts had comments, leaving us with 8310 comment threads for further analysis.

### Measures

#### Attitude Toward COVID-19 Vaccines: Google Cloud Natural Language AI

We used Google Cloud Natural Language AI, a machine learning-based natural language–understanding tool to retrieve the public's attitude toward the COVID-19 vaccine by (1) identifying all entities that were discussed in a given post or comment, (2) using COVID-19 vaccine keywords to distill entities specifically related to the COVID-19 vaccine (eg, COVID-19 vaccine, Pfizer Booster, etc; see Table S3 in Multimedia Appendix 1 for a full list), and (3) measuring attitudes toward each COVID-19 vaccine–related entity. We extracted 23,636 distinct entities from fact-checking posts and 71,418 entities from the comments [20]. To identify entities specifically related to COVID-19 vaccines (eg, Pfizer), as opposed to off-topic entities (eg, President Donald Trump), we only retained entities containing vaccine-related keywords (Multimedia Appendix 1, Table S3). We retrieved 3014 distinct entities in the posts and 3641 entities in the comments that were related to COVID-19 vaccines.

Specifically, we focused on 3 dimensions of the attitude: COVID-19 vaccine entity salience (ie, the salience of COVID-19 vaccine–related entities of all entities in a given text), attitudinal valence (ie, the positive or negative attitude toward vaccine-related entities), and attitudinal magnitude of each COVID-19 vaccine entity (ie, how strong the attitude is).

COVID-19 vaccine *entity salience* is the extent to which COVID-19 vaccine–related entities are discussed in texts relative to discussions of entities that veer off the topic of COVID-19 vaccines; it reflects the importance of vaccine entities in posts/comments (min.=0, max.=1) [20]. For example, a comment exchange might begin with a single comment about Pfizer vaccine misinformation and then veer off the topic of vaccines to a lengthy debate about voter fraud in the US presidential election; in this case, the extent to which a COVID-19 vaccine–related entity (ie, the Pfizer vaccine) was discussed would be relatively low compared to the extent to which off-topic entities (eg, former President Trump) were.

COVID-19 vaccine *attitudinal valence* was operationalized as a sentiment score provided by Google Cloud Natural Language AI, which indicates the overall emotional and attitudinal valence of a text toward a specific entity, ranging from –1 (extremely negative) to 1 (extremely positive), with 0 representing a neutral attitude [20]. Although emotional valence measures the difference between the positive and negative emotions in texts, it does not capture discrete positive and negative emotions in texts. In other words, texts that are considered sad or anxious would both be considered negatively valenced texts.

COVID-19 vaccine *attitudinal magnitude* was operationalized as a separate magnitude score that indicates the extent to which a text is emotionally charged, ranging from 0 (neutral) to positive infinity (extremely emotional) [20]. In other words, unlike attitudinal valence, attitudinal magnitude is nonnormalized and each emotionally valenced expression within a given text (irrespective of the direction of the emotional valence) contributes to the attitudinal magnitude revolving around an entity.

Inferences can be made regarding attitude by combining these 2 dimensions. For example, a comment thread that yields a close-to-zero attitudinal valence and close-to-zero attitudinal magnitude indicates that the thread is unemotional. However, a comment thread that yields a close-to-zero attitudinal valence but high attitudinal magnitude suggests the existence of both highly positive and highly negatively valenced attitudes in the comments that cancel each other out [20].

#### Social Media Engagement

**Figure 2:**
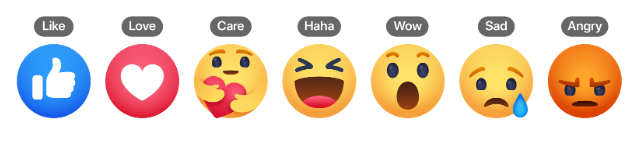
Facebook reaction emoticons.

Social media engagement was operationalized as the number of comments and shares each post received, as well as the number of Facebook reactions a given post received. Facebook reactions are a series of 6 emoticons that enable users to express their emotional responses to posts [38]. See Figure 2 for an overview.

#### Discrete Emotions and Linguistic Features: IBM Watson Tone Analyzer

We used IBM Watson Tone Analyzer, a classifier of discrete emotions and linguistic features based on cognitive linguistic analysis to extract discrete emotions and linguistic features. In contrast to Google Cloud Natural Language AI [20], IBM Watson Tone Analyzer captures specific positive and negative emotions in a given text (ie, joy, anger, fear, sadness) [39]. Regarding linguistic features, we extracted the levels of confidence, tentativeness, and analytical thinking in texts. These variables of discrete emotions and linguistic features range from 0 to 1, with a larger value representing a stronger existence of an attribute.

### Ethics Consideration

This study did not involve human subjects and therefore did not need an institutional review board (IRB) review. The data involved was public data with no identifiable information [40].

## Results

### Analytical Strategy

To answer RQ1 on the changes of the public's attitude toward the COVID-19 vaccine, we aggregated the COVID-19 vaccine fact-checking posts by time and conducted a series of correlation tests between the attitude variables (ie, vaccine entity salience, attitudinal valence, and magnitude) and time.

To answer RQ2 on the effects of different COVID-19 vaccine fact-checking information sources, we conducted multiple linear regressions with the public's attitude toward the COVID-19 vaccine as the dependent variable and negative binomial regressions with social media engagement as the dependent variable. In these regression models, the word count in the posts and comments, discrete emotions and linguistic features in the posts, and Facebook page followers were controlled.

To answer RQ3 and RQ4 on the changes in discrete emotions and linguistic features over time, we conducted a series of correlation tests between discrete emotions, linguistic features, and time.

### The Prevalence of COVID-19 Vaccine Information on Social Media

**Figure 3:**
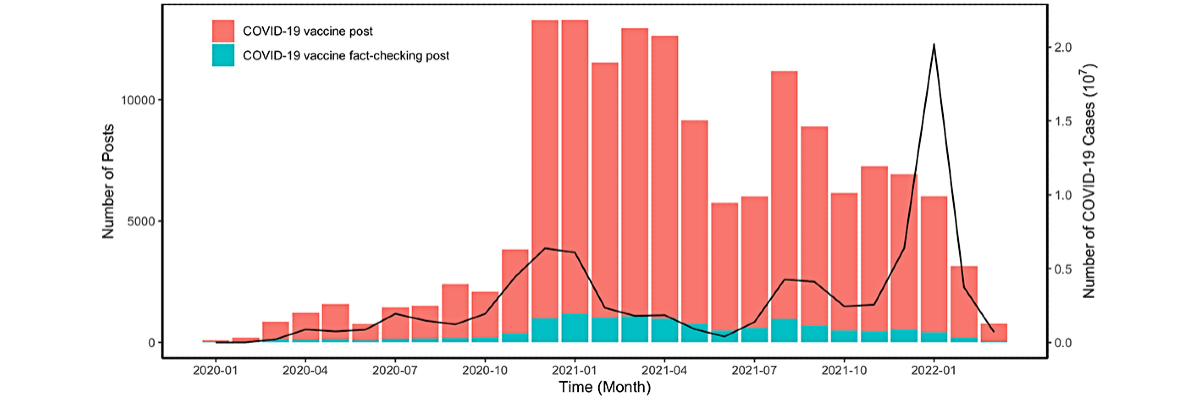
Number of COVID-19 vaccine fact-checking Facebook posts compared to all COVID-19 vaccine–related posts aggregated by month, from January 1, 2020, to March 10, 2022. The line represents the number of new COVID-19 cases, aggregated by month, from January 1, 2020, to March 10, 2022.

**Figure 4:**
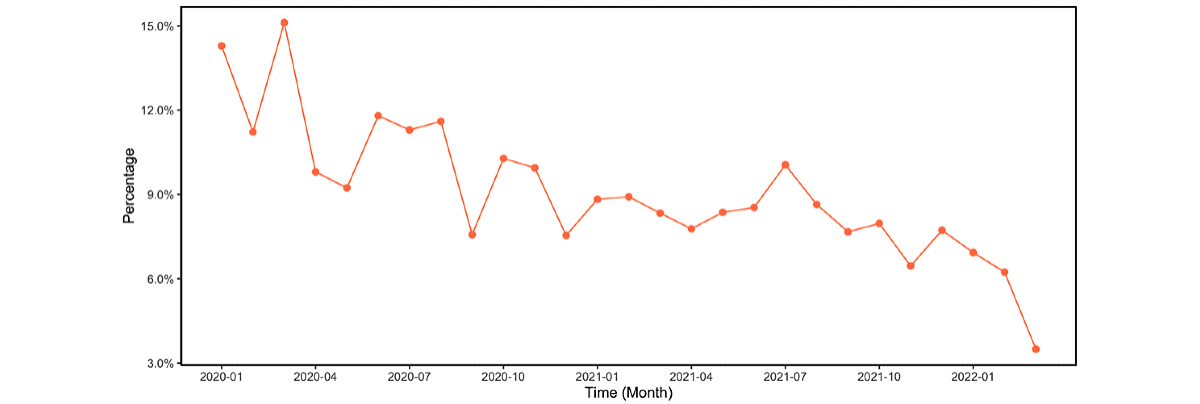
Changes in the percentage of COVID-19 vaccine fact-checking Facebook posts in all COVID-19 vaccine–related Facebook posts over time, aggregated by month, from January 1, 2020, to March 10, 2022.

The number and percentage of COVID-19 fact-checking posts are shown in Figures 3 and 4, respectively. Notably, the percentage of fact-checking posts relative to all COVID-19 vaccine posts steadily decreased as the pandemic progressed (Figure 4).

**Figure 5:**
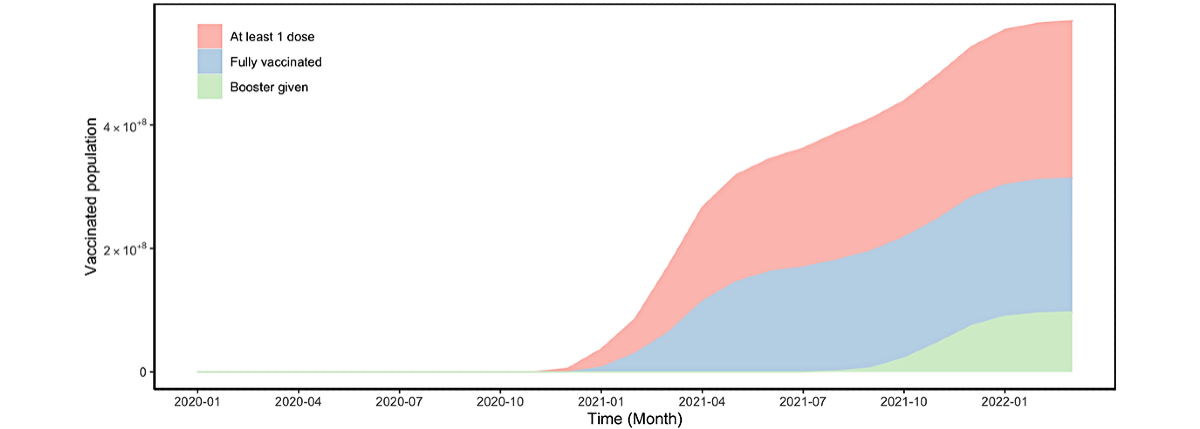
Vaccination population in the United States over time: the number of individuals receiving at least 1 dose, the number of fully vaccinated individuals, and the number of booster shots issued, aggregated by month, from January 1, 2020, to March 10, 2022.

Interestingly, we found that the 2 peaks of the COVID-19 vaccine posts corresponded with the key time points of COVID-19 vaccination (Figures 3 and 5). This pattern was shown by the number of COVID-19 and fact-checking posts peaking in December 2020, when the first COVID-19 vaccines were available to the public. In addition, the number of posts experienced another major soar in August 2021, when the public started to receive COVID-19 booster shots.

### Attitude Toward COVID-19 Vaccine Fact-Checking Posts and Comments Over Time

**Figure 6:**
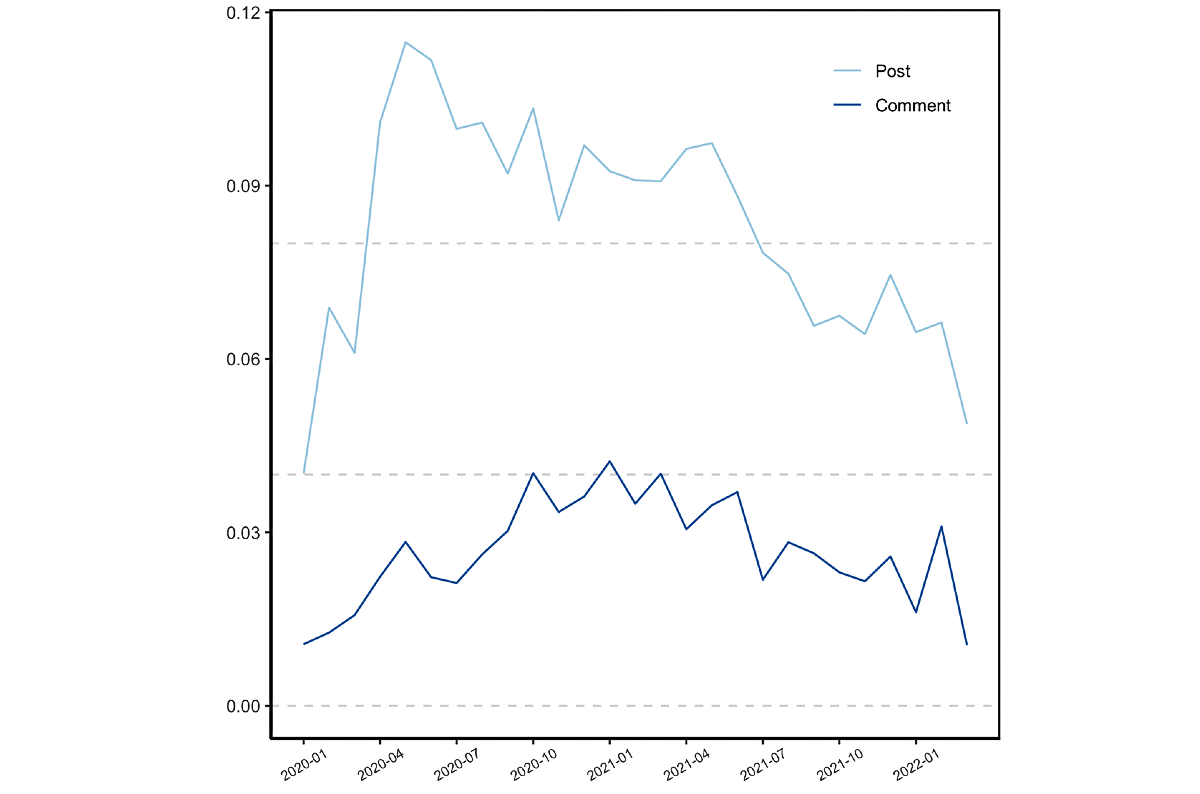
Salience of COVID-19 vaccine–related entities over time, aggregated by month, from January 1, 2020, to March 10, 2022.

To answer RQ1, we aggregated vaccine entity salience and attitudinal valence and magnitude by month. The salience of COVID-19 vaccine–related entities relative to off-topic entities is depicted in Figure 6. The salience of COVID-19 vaccine entities was greater in fact-checking posts (mean 0.09, SD 0.11) compared to comments (mean 0.03, SD 0.09, *t*=39.28, *P*<.001). COVID-19 vaccine salience in the posts started to increase and peaked around May 2020 and continued to decrease since then (*r*=–0.92, *df*=21, *t*=–10.94, 95% CI –0.97 to –0.82, *P*<.001). This may reflect less public concern over vaccine misinformation relative to other COVID-19 topics over time.

**Figure 7:**
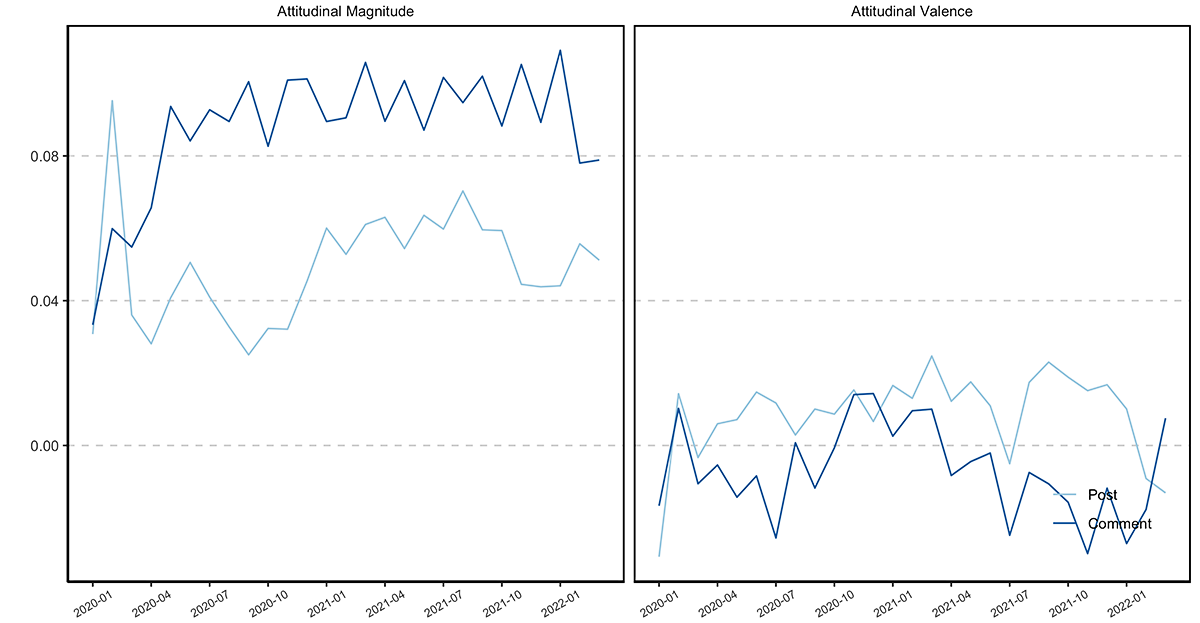
COVID-19 vaccine attitudinal valence and magnitude in COVID-19 vaccine fact-checking posts and comments aggregated by month, from January 1, 2020, to March 10, 2022.

The mean of COVID-19 vaccine attitudinal valence in posts (mean 0.01, SD 0.10) and comments (mean –0.004, SD 0.12) was close to 0. Since the average attitudinal magnitude of posts (mean 0.05, SD 0.11) and comments (mean 0.09, SD 0.13) was close to 0, results revealed that the texts were relatively neutral. Notably, COVID-19 vaccine attitudinal magnitude in comments increased during the pandemic (*r*=0.52, *df*=25, *t*=3.02, 95% CI 0.17-0.75, *P*<.001), which indicates that the public's attitude toward COVID-19 vaccines was becoming more extreme (Figure 7).

Therefore, we noticed a decrease in vaccine entity salience in posts over time (RQ1a) and an increase in attitudinal magnitude in comments over time (RQ1b). Attitudinal valence did not change significantly over time for both posts and comments.

### Information Sources of COVID-19 Vaccine Fact-Checking Posts

**Figure 8:**
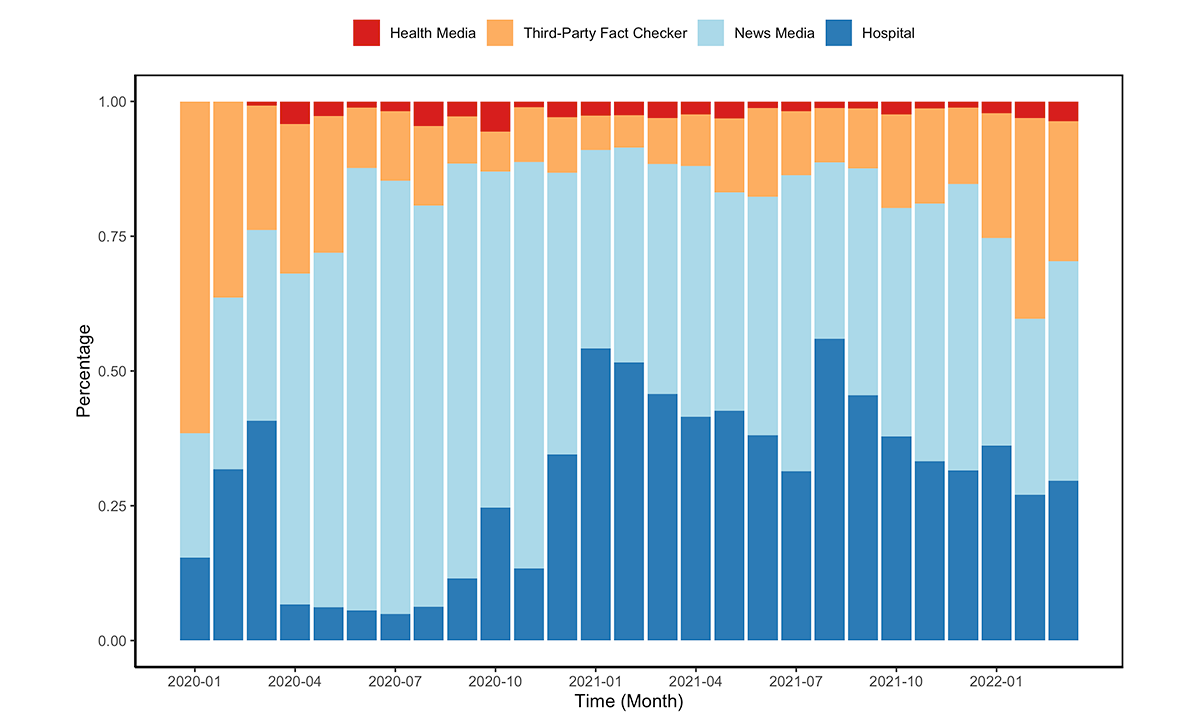
Percentage of COVID-19 vaccine fact-checking Facebook posts by 4 information sources in the United States, aggregated by month, from January 1, 2020, to March 10, 2022.

The majority of COVID-19 vaccine fact-checking Facebook posts were generated by news media (n=5821, 46.4%) and hospitals (n=4921, 39.2%), while relatively fewer posts were posted by third-party fact checkers (n=1523, 12.1%) and US health media (n=288, 2.3%); see Table 1 and Figure 8. Notably, third-party fact checkers have been playing a more important role in more fact-checking over time (*r*=0.63, *df*=25, *t*=4.06, 95% CI 0.33-0.82, *P*<.001).

Social media engagement was operationalized as 9 different metrics, namely, the number of comments (mean 146.76, SD 561.07, median 9) and shares each post received (mean 92.28, SD 594.93, median 8), as well as the number of Facebook reactions a given post received (see Table 2 for an overview). Different information sources have different levels of popularity and social media engagement (Table 3).

Health media have more followers than other 3 sources, while hospitals on average have the least number of followers. COVID-19 vaccine fact-checking posts created by news media were most popular, with the highest number of likes, comments, and shares on average, and posts by hospitals were least popular.

**Table 1:** Summary statistics for sources of COVID-19 fact-checking posts.

Information source	Facebook pages (N=2644), n (%)	Posts (N=12,553), n (%)
News media	95 (3.6)	5821 (46.4)
Hospitals	1096 (41.5)	4921 (39.2)
Third-party fact checkers	9 (0.3)	1523 (12.1)
US health media	26 (1.0)	288 (2.3)

**Table 2:** Summary statistics for Facebook reactions.

Reaction metric	Mean (SD)	Median
Like	427.65 (1946.95)	41
Love	42 (346.86)	1
Wow	26.04 (199.94)	0
Haha	64.96 (406)	2
Sad	26.95 (218.49)	0
Angry	76.33 (554.58)	0
Care	3.96 (19.84)	0

**Table 3:** Average social media engagement of COVID-19 vaccine fact-checking Facebook posts across information sources.

Category	Third-party fact checker	Health media	Hospital	News media
Average followers	538,028	2,193,198	15,639	6,619,852
Posts (N=12,553), n (%)	1523 (12.1)	288 (2.3)	4921 (39.2)	5821 (46.4)
Likes, mean (SD)	231.55 (587.35)	252.07 (1039.86)	24 (162.96)	828.88 (2,774.68)
Comments, mean (SD)	125.1 (228.83)	144.38 (320)	9.39 (150.71)	268.67 (781.04)
Shares, mean (SD)	100.14 (476.54)	125.02 (398.62)	27.07 (650.73)	143.72 (576.06)
Love, mean (SD)	7.66 (58.81)	10.77 (31.86)	3.34 (33.04)	85.22 (504.1)
Wow, mean (SD)	9.7 (33.28)	10.3 (44.35)	0.16 (1.62)	52.96 (290.61)
Haha, mean (SD)	50.08 (184.47)	33.14 (89.59)	1.32 (42.64)	124.23 (581.05)
Sad, mean (SD)	10.67 (42.26)	8.4 (38.94)	0.5 (6.86)	54.49 (317.71)
Angry, mean (SD)	43.56 (194.11)	10.24 (32.22)	0.51 (10.83)	152.27 (801.37)
Care, mean (SD)	1.2 (5.91)	2.75 (10.14)	0.98 (11.66)	7.26 (26.45)

### Information Sources and COVID-19 Vaccine Attitude

To answer RQ2a, we conducted multiple linear regressions to investigate the effects of information sources on COVID-19 vaccine attitude, with word counts in the posts and comments and the number of Facebook page followers included as control variables (Multimedia Appendix 1, Tables S13-S15). Results showed that the public's attitudinal valence on COVID-19 vaccines significantly increased with fact-checking posts from hospitals (b=0.06, 95% CI 0.00-0.12, *t*=2.03, *P*=.04), though we found no significant effects of information sources on the salience or attitudinal magnitude related to COVID-19 vaccines in the comments. This suggested that hospitals' fact-checking posts on Facebook significantly improved the public's valence attitude toward COVID-19 vaccines.

### Information Sources and Social Media Engagement

To answer RQ2b, we aggregated fact-checking posts by Facebook page information source and created negative binomial regression models to assess whether the type of information source significantly predicted the 9 metrics of social media engagement, while controlling for post word count and follower count (Multimedia Appendix 1, Tables S4-S12).

**Figure 9:**
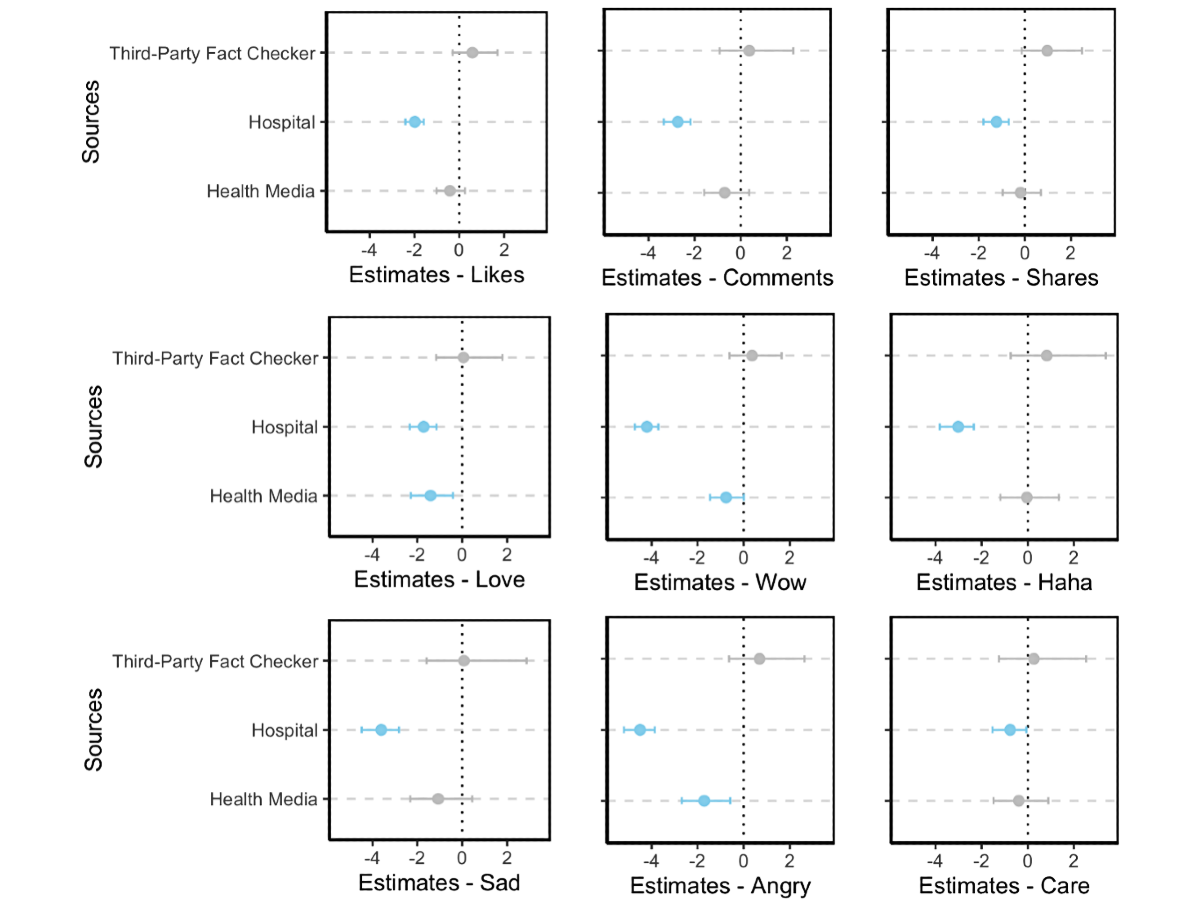
Regression coefficients (95% CI) of information sources with significant effects on social media engagement in negative binomial models. Blue dots and blue error bars show significant coefficients and 95% CIs (*P*>.05); gray dots and gray error bars show insignificant coefficients and 95% CIs (*P*>.05). Exact coefficients and *P* values can be accessed in Multimedia Appendix 1, Tables S4-S12.

Results revealed that hospitals have a significantly lower social media engagement for all engagement metrics than news media. Similarly, health media had significantly fewer wow and angry reactions than news media (*P*>.05; Figure 9). Results revealed that hospitals also have a smaller audience than other sources of health information. Additionally, although health media and news media posts had similar levels of engagement, health media evoked fewer wow and angry reactions from the public.

### Emotional Trends in COVID-19 Vaccine Fact-Checking Posts and Comments

**Figure 10:**
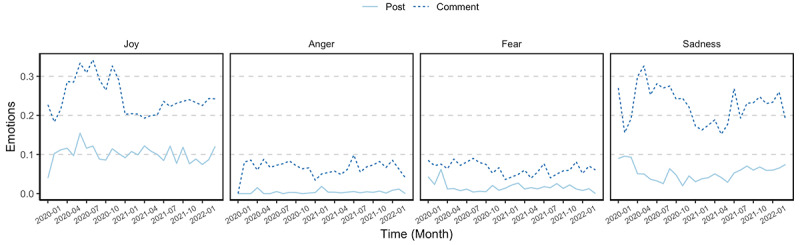
Trends in discrete emotions in COVID-19 vaccine fact-checking posts and comments, aggregated by month, from January 1, 2020, to March 10, 2022.

To answer RQ3, we used IBM Watson Tone Analyzer to extract 4 discrete emotions and 3 linguistic features in COVID-19 vaccine fact-checking posts and comments (Figure 10) [39]. Findings revealed that comments were more emotionally charged than fact-checking posts in terms of joy (mean_post_ 0.10, SD_post_ 0.23, mean_comment_ 0.23, SD_comment_ 0.30, *t*=–38.90), sadness (mean_post_ 0.05, SD_post_ 0.16, mean_comment_ 0.21, SD_comment_ 0.28, *t*=–56.16), anger (mean_post_ 0.01, SD_post_ 0.05, mean_comment_ 0.06, SD_comment_ 0.18, *t*=–34.53), and fear (mean_post_ 0.02, SD_post_ 0.10, mean_comment_ 0.06, SD_comment_ 0.17, *t*=–21.96, *P*<.001). This suggests that fact-checking posts tended to maintain a neutral tone, whereas the public comments were more emotionally charged. Results also revealed that the dominant emotions in both comments and posts were joy and sadness.

### Linguistic Features in COVID-19 Vaccine Fact-Checking Posts and Comments

**Figure 11:**
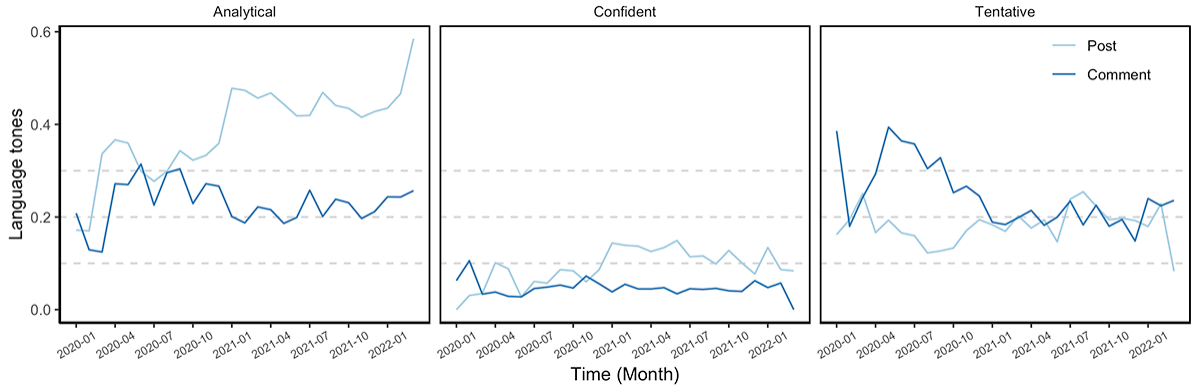
Changes in linguistic features in COVID-19 vaccine fact-checking posts and comments, aggregated by month, from January 1, 2020, to March 10, 2022.

To answer RQ4, we used IBM Watson Tone Analyzer to extract 3 linguistic feature attributes of COVID-19 vaccine fact-checking posts and comments, namely post analytical thinking, confidence, and tentativeness (Figure 11) [39]. Fact-checking posts were more analytical (mean_post_ 0.43, SD_post_ 0.40, mean_comment_ 0.22, SD_comment_ 0.33, *t*=44.09), more confident (mean_post_ 0.11, SD_post_ 0.28, mean_comment_ 0.05, SD_comment_ 0.19, *t*=22.50), and less tentative (mean_post_ 0.19, SD_post_ 0.34, mean_comment_ 0.21, SD_comment_ 0.34, *t*=–5.19, *P*<.001) than in the comments.

In addition, linguistic features changed over time (Figure 11). COVID-19 vaccine fact-checking posts continued to be more analytical (*r*=0.81, *df*=25, *t*=6.88, 95% CI 0.62-0.91, *P*<.001) and more confident (*r*=0.59, *df*=25, *t*=3.68, 95% CI 0.27-0.79, *P*=.001) over time. This suggests that as public health officials gained more information about the COVID-19 vaccine, they expressed heightened confidence in and reduced tentativeness about the vaccine.

Although comments did not exhibit a significant increase in confidence over time, tentativeness in comments decreased significantly (*r*=–0.62, *df*=25, *t*=–3.94, 95% CI –0.81 to –0.31, *P*=.001). Results suggested that both public health officials and the public expressed more certain attitudes, in terms of tentativeness and confidence, toward the vaccine as more information became available.

### Discrete Emotions and Linguistic Features by Information Sources

**Figure 12:**
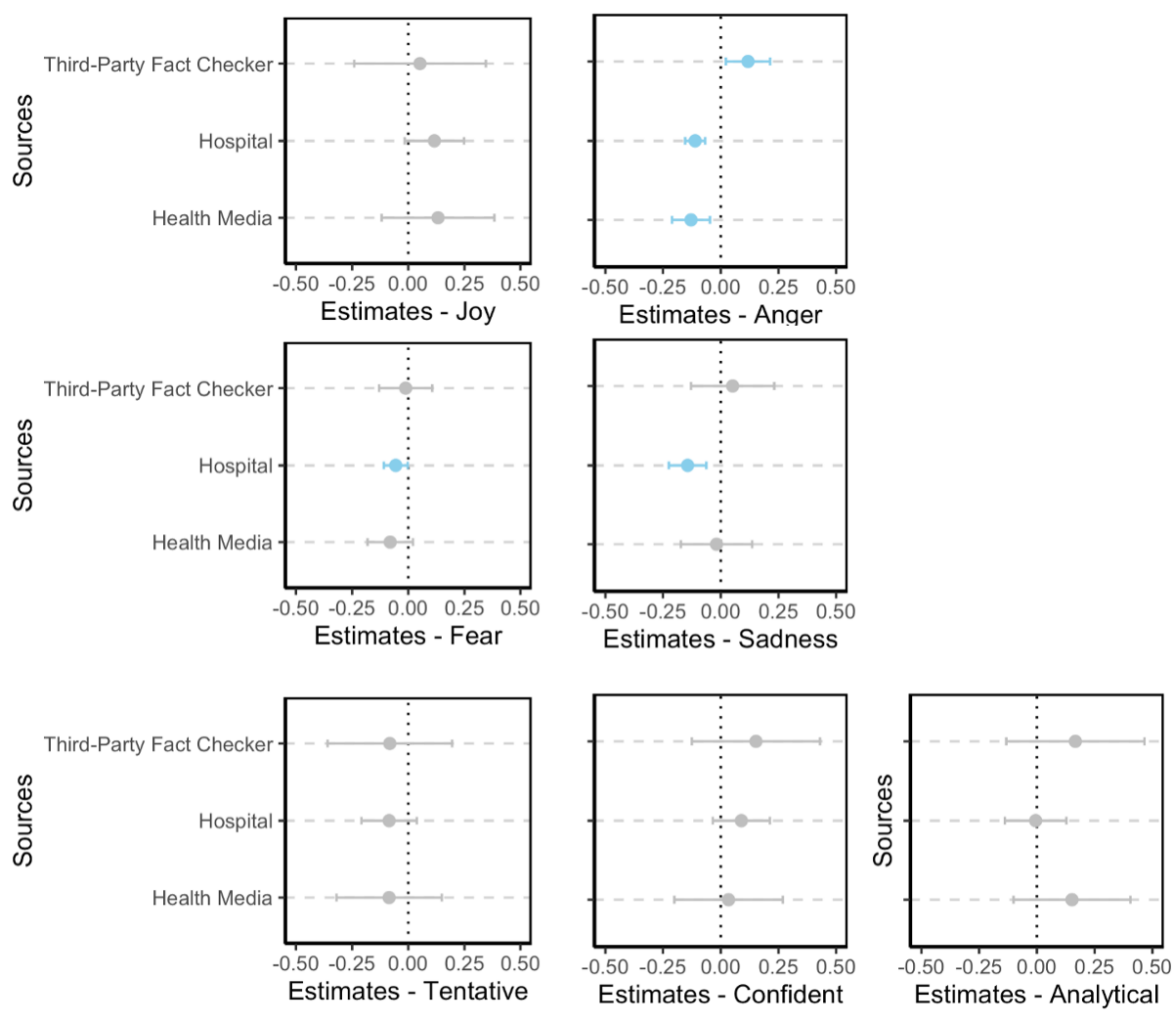
Regression coefficients (95% CIs) of information sources with significant effects on emotions and language tones in comments in linear regression models. Blue dots and blue error bars show significant coefficients and 95% CIs (*P*<.05); gray dots and gray error bars show insignificant coefficients and 95% CIs (P≥.05). Actual coefficients and *P* values can be accessed in Multimedia Appendix 1, Tables S16-S22.

In addition to the proposed RQs, we explored how comments might respond differently across information sources regarding discrete emotions and linguistic features with multiple linear regression analyses. The word count of posts and comments, the Facebook page follower count, and discrete emotions and linguistic features of posts were controlled (Multimedia Appendix 1, Tables S16-S19). Regression coefficients are shown in Figure 12.

Results revealed that fact-checking posts from hospitals were associated with lower levels of anger, fear, and sadness in comments, while posts from third-party fact checkers were associated with heightened comment anger. In other words, third party fact checkers tended to evoke heightened comment anger, whereas comments on posts from health media and hospitals expressed less negative emotion. However, no significant effects were found for linguistic features.

## Discussion

### Principal Findings

This study examined the US COVID-19 vaccine fact-checking information on Facebook and analyzed the effects of different fact-checking posts’ information sources on the public's attitude toward COVID-19 vaccines and social media engagement. We observed the prevalence and trend of COVID-19 vaccine fact-checking information on Facebook. Findings revealed health information Facebook pages responded to the COVID-19 infodemic by posting most frequently at 2 key vaccine time points in the United States: (1) when the vaccine first became available in December 2020 and (2) when the booster shot became available in August 2021 [41].

Notably, the percentage of fact-checking posts relative to all COVID-19 vaccine posts steadily decreased as the pandemic progressed. This may be because the frequency of COVID-19 vaccine posts increased at a higher rate than fact-checking vaccine posts. Another explanation is that public health organizations’ efforts to promote accurate COVID-19 vaccine information reduced COVID-19 vaccine misinformation, necessitating that users fact-check misinformation. Likewise, posts tended to focus more on COVID-19 vaccine entities than comments did, reflecting the public concern over a more diverse set of topics relative to the vaccine itself. This may be because as the pandemic progressed, Facebook and sources of health information took actions to mitigate vaccine misinformation, necessitating less misinformation corrections and vaccine discourse over time. For example, Facebook began removing COVID-19 health misinformation and attaching various warnings to misleading posts, and public health initiatives promoted accurate COVID-19 vaccine information [42,43].

### The Role of Hospitals in Communicating COVID-19 Vaccine Information

Our most prominent finding was that hospitals play a key role in disseminating facts and correcting misinformation. Although hospitals receive less engagement than other information sources, the comments expressed more positive emotions compared to other information sources. This suggests that hospitals should invest more in generating engaging public health campaigns on social media.

Regarding overall emotions in the comments, fact-checking posts from health media and hospitals were associated with lower levels of anger, fear, and sadness in the comments, while posts from third-party fact checkers were associated with higher levels of anger in the comments. These negative emotions are crucial heuristic cues to the public's attitude and therefore should be acknowledged by information and health influencers in communicating facts and correcting misinformation. Empathetic communication enables fact-checking practitioners to better connect with the audience and counterbalance the negative emotions and hesitancy evoked by COVID-19 vaccine misinformation [25].

In addition, the majority of COVID-19 vaccine fact-checking Facebook posts were generated by news media and hospitals, while relatively few were from third-party fact checkers and US health media. Notably, third-party fact checkers posted more COVID-19 vaccine posts as the pandemic progressed. Although health media were the smallest source COVID-19 vaccine fact-checking posts, they have more followers than the other 3 sources, and although hospitals generated more fact-checking posts, they have the fewest followers. Although health media posts had similar levels of engagement as news media, they elicited few wow and angry reactions, likely reflecting a less negative attitude amongst followers of health media compared to news media. This may be because news media communicate with the general public, while health-concerned people follow health media and tend to have consistent health views. COVID-19 vaccine fact-checking posts created by news media were most popular, with the highest number of likes, comments, and shares on average, whereas users engaged with posts from hospitals the least.

### Evolution of the Public's Attitude Over Time

Posts and comments tended to be relatively neutral in nature with low levels of attitudinal valence. However, as the pandemic progressed, the salience of COVID-19 vaccine entities in posts kept decreasing, and the publics’ comments became more extreme, with higher levels of attitudinal magnitude. This suggests that fact-checking posts tend to report news and communicate facts objectively and have shifted the focus from the COVID-19 vaccine itself to other related subjects. However, the public’s attitude became increasingly extreme over time. This supports extant findings that early interventions, such as inoculation against misinformation before attitude becomes increasingly extreme, may be more effective in the long term [44].

In addition, the salience of COVID-19 vaccine entities was significantly lower in comments than in posts. This suggested that the public is more concerned with issues other than the COVID-19 vaccine. The discrepancy in posts and comments further suggests the need for responsive and empathetic communication that might be more effective in improving the vaccine attitude and confidence.

### Discrete Emotions and Linguistic Features

In line with our conclusion that public comments became more extreme as the pandemic progressed, fact-checking comments exhibited heightened joy, anger, fear, and sadness than posts. Although the presence of heightened positive emotions (eg, joy) in COVID-19 health messages has shown to predict compliance with COVID-19 public health guidelines [26], it is also true that individuals who have a positive attitude toward the vaccine may opt to generate and seek out COVID-19 misinformation corrections to reinforce their positive attitude [45]. Thus, these messages may not be reaching vaccine-hesitant individuals.

Likewise, users may seek and engage with sad content to manage negative emotions [45]. Notably, just as sadness can protect against initial belief in misinformation [46], it also seems to facilitate attitude change when encountered by misinformation corrections [47]. Thus, heightened sadness in fact-checking messages may hold promise for mitigating vaccine hesitancy.

Emotions and linguistic features in both COVID-19 vaccine fact-checking posts and comments evolved over time. The posts adopted a more analytical and confident tone over time, while we observed a significant drop in fear and tentativeness in the comments. Both trends suggest that with more information we know about the pandemic and the COVID-19 vaccine, the confidence related to the COVID-19 vaccine increases for both information sources and the general public.

### Limitations

Although our findings shed light on COVID-19 vaccine fact-checking in a naturalistic setting, this study is not without limitations. By focusing on a sample of US posts, we neglected to explore how fact-checking manifests in other countries. Additionally, as different platforms have different behavioral norms [48], it is reasonable for user behaviors to vary by platform. Furthermore, our natural language processing sentiment analysis tools do not allow for the more refined coding established by human coders. However, we used a machine learning approach, which has shown to yield increased explanatory power, to reduce this limitation [49]. Relatedly, as with all observational studies, we cannot infer the direction of causality. Lastly, Facebook data usage restrictions prohibit researchers from collecting all comments; however, researchers are permitted to mine the top 25 comments for posts. The more user engagement (ie, likes, reactions, replies) a comment has, the higher up Facebook algorithmically ranks it in the comment thread [50]; thus, although we acknowledge not having all comments as a limitation, we believe the top 25 comments for each post are sufficiently informative and representative of public opinion.

### Conclusion

This study has broad implications for public health practitioners and social media managers. First, although hospitals play a large role in fact-checking COVID-19 vaccine misinformation, they should work to design posts that will better engage the public. Hospitals are perceived as credible information sources with authority as health institutions, which makes them credible sources that are highly likely to elicit attitude and behavior change on health issues. Second, as fact-checking posts evoked increasingly extreme public attitude over time, early interventions (ie, social media campaigns that inoculate against misinformation before it becomes mainstream) are critical. Additionally, fact-checking information sources should engage in empathetic communication to better address the concerns of the public and empathize with the public. For example, sadness can both protect against belief in misinformation and facilitate attitude change when confronted with misinformation corrections [46,47]. Expression of sadness in fact-checking messages holds promise for mitigating vaccine hesitancy. Distinct emotions are crucial heuristic cues for public attitude formation and therefore should be acknowledged by social media managers tasked with communicating facts and correcting misinformation, and, ultimately, countering COVID-19 vaccine hesitancy [25]. Finally, in contrast to health media and hospitals, who evoked less anger in public responses to fact-checking COVID-19 vaccine posts, third-party fact checkers tended to evoke heightened anger in responses; fact-checking agencies should be mindful of this when communicating with the public.

## Appendix

Multimedia Appendix 1 Supplemental material.

## Abbreviations

AI: artificial intelligence
RQ: research question
