# Supplementary material for: COVID-19 Vaccine Fact-Checking Posts on Facebook: Observational Study
Source: J Med Internet Res. 2022 Jun 21;24(6):e38423. doi: 10.2196/38423 (PMC9217154; doi:10.2196/38423)
Supplement: Multimedia Appendix 1 [file jmir_v24i6e38423_app1.docx]

**Multimedia Appendix 1. Supplemental Material**

Table S1. Keyword combinations to identify COVID-19 vaccine-related Facebook posts.

| covid vaccine | coronavirus vaccine | biontech vaccine | johnson and johnson vaccine | moderna vaccine | attenuated vaccine |
| --- | --- | --- | --- | --- | --- |
| covid shot | coronavirus shot | biontech shot | johnson and johnson shot | moderna shot | attenuated shot |
| covid jab | coronavirus jab | biontech jab | johnson and johnson jab | moderna jab | attenuated jab |
| covid booster | coronavirus booster | biontech booster | johnson and johnson booster | moderna booster | attenuated booster |
| covid dose | coronavirus dose | biontech dose | johnson and johnson dose | moderna dose | attenuated dose |
| covid injection | coronavirus injection | biontech injection | johnson and johnson injection | moderna injection | attenuated injection |
| covid-19 vaccine | pfizer vaccine | johnson & johnson vaccine | j&j vaccine | niaid vaccine | mrna vaccine |
| covid-19 shot | pfizer shot | johnson & johnson shot | j&j shot | niaid shot | mrna shot |
| covid-19 jab | pfizer jab | johnson & johnson jab | j&j jab | niaid jab | mrna jab |
| covid-19 booster | pfizer booster | johnson & johnson booster | j&j booster | niaid booster | mrna booster |
| covid-19 dose | pfizer dose | johnson & johnson dose | j&j dose | niaid dose | mrna dose |
| covid-19 injection | pfizer injection | johnson & johnson injection | j&j injection | niaid injection | mrna injection |

Table S2. Keyword combinations to identify COVID-19 vaccine-related fact-checking Facebook posts.

| factcheck | fact-check | fact check | misinformation | fake news | rumor |  |
| --- | --- | --- | --- | --- | --- | --- |
| debunk | myth | hoax | disinformation | fact | false | true |

Table S3. Vaccine-related keywords to identify COVID-19 vaccine-related entities.

| vax | vac | shot | booster | jab | dose |
| --- | --- | --- | --- | --- | --- |
| biontech | johnson & johnson | johnson and johnson | j&j | moderna | niaid |
| pfizer |  |  |  |  |  |

Table S4. Negative binomial regression model of information sources on the average number of likes per Facebook page.

| Likes | Estimate | SE | Lower 95% CI | Upper 95% CI | p |
| --- | --- | --- | --- | --- | --- |
| Intercept | 4.81 | 0.16 | 4.42 | 5.23 | <.001*** |
| Health Media | -0.42 | 0.31 | -1.02 | 0.25 | .181 |
| Hospital | -1.98 | 0.17 | -2.41 | -1.58 | <.001*** |
| Third-party Fact Checker | 0.58 | 0.49 | -0.29 | 1.70 | .233 |
| Post Word Count | 0.002 | <.001 | 0.002 | 0.003 | <.001*** |
| Page Follower | <.001 | <.001 | <.001 | <.001 | <.001*** |

*** p < .001; ** p < .01; * p < .05; . p < .10

Table S5. Negative binomial regression model of information sources on the average number of comments per Facebook page.

| Comments | Estimate | SE | Lower 95% CI | Upper 95% CI | p |
| --- | --- | --- | --- | --- | --- |
| Intercept | 4.24 | 0.25 | 3.69 | 4.85 | <.001*** |
| Health Media | -0.69 | 0.49 | -1.59 | 0.37 | .155 |
| Hospital | -2.73 | 0.26 | -3.34 | -2.18 | <.001*** |
| Third-party Fact Checker | 0.37 | 0.77 | -0.92 | 2.28 | .631 |
| Post Word Count | 0.003 | 0.001 | 0.002 | 0.004 | <.001*** |
| Page Follower | <.001 | <.001 | <.001 | <.001 | <.001*** |

Table S6. Negative binomial regression model of information sources on the average number of shares per Facebook page.

| Shares | Estimate | SE | Lower 95% CI | Upper 95% CI | p |
| --- | --- | --- | --- | --- | --- |
| Intercept | 3.33 | 0.21 | 2.81 | 3.89 | <.001*** |
| Health Media | -0.18 | 0.40 | -0.96 | 0.69 | .651 |
| Hospital | -1.23 | 0.22 | -1.80 | -0.70 | <.001*** |
| Third-party Fact Checker | 0.97 | 0.63 | -0.14 | 2.48 | .124 |
| Post Word Count | 0.007 | <.001 | 0.006 | 0.008 | <.001*** |
| Page Follower | <.001 | <.001 | <.001 | <.001 | <.001*** |

Table S7. Negative binomial regression model of information sources on the average number of love per Facebook page.

| Love | Estimate | SE | Lower 95% CI | Upper 95% CI | p |
| --- | --- | --- | --- | --- | --- |
| Intercept | 2.44 | 0.23 | 1.89 | 3.04 | <.001*** |
| Health Media | -1.41 | 0.46 | -2.29 | -0.41 | .002** |
| Hospital | -1.72 | 0.24 | -2.34 | -1.15 | <.001*** |
| Third-party Fact Checker | 0.06 | 0.71 | -1.17 | 1.79 | .937 |
| Post Word Count | 0.005 | <.001 | 0.004 | 0.006 | <.001*** |
| Page Follower | <.001 | <.001 | <.001 | <.001 | <.001*** |

Table S8. Negative binomial regression model of information sources on the average number of wow per Facebook page.

| Wow | Estimate | SE | Lower 95% CI | Upper 95% CI | p |
| --- | --- | --- | --- | --- | --- |
| Intercept | 2.15 | 0.18 | 1.70 | 2.63 | <.001*** |
| Health Media | -0.76 | 0.36 | -1.46 | 0.01 | .032* |
| Hospital | -4.20 | 0.21 | -4.72 | -3.71 | <.001*** |
| Third-party Fact Checker | 0.37 | 0.55 | -0.61 | 1.64 | .507 |
| Post Word Count | 0.001 | 0.001 | <.001 | 0.002 | .115 |
| Page Follower | <.001 | <.001 | <.001 | <.001 | <.001*** |

Table S9. Negative binomial regression model of information sources on the average number of haha per Facebook page.

| Haha | Estimate | SE | Lower 95% CI | Upper 95% CI | p |
| --- | --- | --- | --- | --- | --- |
| Intercept | 2.87 | 0.31 | 2.17 | 3.71 | <.001*** |
| Health Media | -0.04 | 0.61 | -1.19 | 1.35 | .948 |
| Hospital | -3.02 | 0.33 | -3.82 | -2.33 | <.001*** |
| Third-party Fact Checker | 0.82 | 0.96 | -0.74 | 3.38 | .391 |
| Post Word Count | -0.003 | 0.001 | -0.004 | -0.002 | .001** |
| Page Follower | <.001 | <.001 | <.001 | <.001 | <.001*** |

Table S10. Negative binomial regression model of information sources on the average number of sad per Facebook page.

| Sad | Estimate | SE | Lower 95% CI | Upper 95% CI | p |
| --- | --- | --- | --- | --- | --- |
| Intercept | 2.74 | 0.33 | 1.99 | 3.58 | <.001*** |
| Health Media | -1.07 | 0.65 | -2.31 | 0.44 | .099. |
| Hospital | -3.61 | 0.35 | -4.49 | -2.83 | <.001*** |
| Third-party Fact Checker | 0.08 | 1.02 | -1.59 | 2.87 | .938 |
| Post Word Count | 0.002 | 0.001 | <.001 | 0.004 | .018* |
| Page Follower | <.001 | <.001 | <.001 | <.001 | .002** |

Table S11. Negative binomial regression model of information sources on the average number of angry per Facebook page.

| Angry | Estimate | SE | Lower 95% CI | Upper 95% CI | p |
| --- | --- | --- | --- | --- | --- |
| Intercept | 3.35 | 0.26 | 2.74 | 4.04 | <.001*** |
| Health Media | -1.71 | 0.50 | -2.69 | -0.58 | .001** |
| Hospital | -4.50 | 0.27 | -5.20 | -3.86 | <.001*** |
| Third-party Fact Checker | 0.69 | 0.78 | -0.63 | 2.64 | .375 |
| Post Word Count | 0.000 | 0.001 | -0.002 | 0.001 | .580 |
| Page Follower | <.001 | <.001 | <.001 | <.001 | <.001 |

Table S12. Negative binomial regression model of information sources on the average number of care per Facebook page.

| Care | Estimate | SE | Lower 95% CI | Upper 95% CI | p |
| --- | --- | --- | --- | --- | --- |
| Intercept | 0.30 | 0.29 | -0.37 | 1.04 | .308 |
| Health Media | -0.39 | 0.58 | -1.48 | 0.89 | .501 |
| Hospital | -0.76 | 0.31 | -1.53 | -0.06 | .013* |
| Third-party Fact Checker | 0.27 | 0.89 | -1.25 | 2.53 | .765 |
| Post Word Count | 0.005 | 0.001 | 0.004 | 0.007 | <.001*** |
| Page Follower | <.001 | <.001 | <.001 | <.001 | <.001*** |

Table S13. Linear regression model of information sources on the salience of COVID-19 vaccine in the comments.

| Salience | Estimate | SE | Lower 95% CI | Upper 95% CI | p |
| --- | --- | --- | --- | --- | --- |
| Intercept | 0.04 | 0.02 | 0.00 | 0.08 | .050. |
| Health Media | -0.02 | 0.04 | -0.10 | 0.06 | .687 |
| Hospital | 0.01 | 0.02 | -0.03 | 0.05 | .756 |
| Third-party Fact Checker | 0.00 | 0.05 | -0.10 | 0.09 | .936 |
| Post Word Count | <.001 | <.001 | <.001 | <.001 | .941 |
| Comment Word Count | <.001 | <.001 | <.001 | <.001 | .173 |
| Page Follower | <.001 | <.001 | <.001 | <.001 | .965 |

Table S14. Linear regression model of information sources on the attitudinal valence of COVID-19 vaccine in the comments.

| Sentiment Score | Estimate | SE | Lower 95% CI | Upper 95% CI | p |
| --- | --- | --- | --- | --- | --- |
| Intercept | -0.03 | 0.03 | -0.08 | 0.03 | .389 |
| Health Media | 0.01 | 0.06 | -0.10 | 0.12 | .872 |
| Hospital | 0.06 | 0.03 | 0.00 | 0.12 | .043* |
| Third-party Fact Checker | -0.02 | 0.07 | -0.15 | 0.11 | .791 |
| Post Word Count | <.001 | <.001 | <.001 | <.001 | .184 |
| Comment Word Count | <.001 | <.001 | <.001 | <.001 | .832 |
| Page Follower | <.001 | <.001 | <.001 | <.001 | .780 |

Table S15. Linear regression model of information sources on the attitudinal magnitude of COVID-19 vaccine in the comments.

| Sentiment Magnitude | Estimate | SE | Lower 95% CI | Upper 95% CI | p |
| --- | --- | --- | --- | --- | --- |
| Intercept | 0.02 | 0.03 | -0.03 | 0.08 | .389 |
| Health Media | 0.00 | 0.05 | -0.11 | 0.11 | .989 |
| Hospital | 0.03 | 0.03 | -0.02 | 0.09 | .232 |
| Third-party Fact Checker | 0.01 | 0.06 | -0.12 | 0.14 | .863 |
| Post Word Count | <.001 | <.001 | <.001 | <.001 | .594 |
| Comment Word Count | <.001 | <.001 | <.001 | <.001 | <.001*** |
| Page Follower | <.001 | <.001 | <.001 | <.001 | .805 |

Table S16. Linear regression model of information sources on joy in the comments.

| Joy | Estimate | SE | Lower 95% CI | Upper 95% CI | p |
| --- | --- | --- | --- | --- | --- |
| Intercept | 0.15 | 0.07 | 0.02 | 0.29 | .024* |
| Health Media | 0.13 | 0.13 | -0.12 | 0.38 | .299 |
| Hospital | 0.12 | 0.07 | -0.02 | 0.25 | .084. |
| Third-party Fact Checker | 0.05 | 0.15 | -0.24 | 0.34 | .726 |
| Post Word Count | <.001 | <.001 | <.001 | <.001 | .936 |
| Comment Word Count | <.001 | <.001 | <.001 | 0.001 | .001** |
| Page Follower | <.001 | <.001 | <.001 | <.001 | .987 |
| Joy in Posts | 0.28 | 0.08 | 0.13 | 0.43 | <.001*** |
| Anger in Posts | 0.22 | 2.10 | -3.92 | 4.36 | .915 |
| Fear in Posts | 0.08 | 0.21 | -0.33 | 0.48 | .700 |
| Sadness in Posts | 0.12 | 0.13 | -0.13 | 0.38 | .336 |

Table S17. Linear regression model of information sources on anger in the comments.

| Anger | Estimate | SE | Lower 95% CI | Upper 95% CI | p |
| --- | --- | --- | --- | --- | --- |
| Intercept | 0.14 | 0.02 | 0.09 | 0.18 | <.001*** |
| Health Media | -0.13 | 0.04 | -0.21 | -0.05 | .002** |
| Hospital | -0.11 | 0.02 | -0.15 | -0.07 | <.001*** |
| Third-party Fact Checker | 0.12 | 0.05 | 0.02 | 0.21 | .016* |
| Post Word Count | <.001 | <.001 | <.001 | <.001 | .862 |
| Comment Word Count | <.001 | <.001 | <.001 | <.001 | .863 |
| Page Follower | <.001 | <.001 | <.001 | <.001 | .932 |
| Joy in Posts | -0.01 | 0.03 | -0.06 | 0.04 | .653 |
| Anger in Posts | 0.67 | 0.69 | -0.68 | 2.03 | .328 |
| Fear in Posts | -0.01 | 0.07 | -0.14 | 0.12 | .872 |
| Sadness in Posts | -0.06 | 0.04 | -0.15 | 0.02 | .141 |

Table S18. Linear regression model of information sources on fear in the comments.

| Fear | Estimate | SE | Lower 95% CI | Upper 95% CI | p |
| --- | --- | --- | --- | --- | --- |
| Intercept | 0.09 | 0.03 | 0.03 | 0.14 | .002** |
| Health Media | -0.08 | 0.05 | -0.18 | 0.02 | .120 |
| Hospital | -0.06 | 0.03 | -0.11 | -0.002 | .040* |
| Third-party Fact Checker | -0.01 | 0.06 | -0.13 | 0.11 | .847 |
| Post Word Count | <.001 | <.001 | <.001 | <.001 | .554 |
| Comment Word Count | <.001 | <.001 | <.001 | <.001 | .390 |
| Page Follower | <.001 | <.001 | <.001 | <.001 | .818 |
| Joy in Posts | -0.03 | 0.03 | -0.09 | 0.04 | .418 |
| Anger in Posts | 0.45 | 0.85 | -1.22 | 2.12 | .598 |
| Fear in Posts | -0.06 | 0.08 | -0.22 | 0.11 | .502 |
| Sadness in Posts | 0.04 | 0.05 | -0.06 | 0.14 | .450 |

Table S19. Linear regression model of information sources on sadness in the comments.

| Sadness | Estimate | SE | Lower 95% CI | Upper 95% CI | p |
| --- | --- | --- | --- | --- | --- |
| Intercept | 0.21 | 0.04 | 0.13 | 0.29 | <.001 |
| Health Media | -0.02 | 0.08 | -0.17 | 0.14 | .817 |
| Hospital | -0.14 | 0.04 | -0.22 | -0.06 | .001** |
| Third-party Fact Checker | 0.05 | 0.09 | -0.13 | 0.23 | .573 |
| Post Word Count | <.001 | <.001 | <.001 | <.001 | .964 |
| Comment Word Count | <.001 | <.001 | <.001 | 0.001 | <.001*** |
| Page Follower | <.001 | <.001 | <.001 | <.001 | .553 |
| Joy in Posts | 0.00 | 0.05 | -0.10 | 0.09 | .926 |
| Anger in Posts | -0.05 | 1.29 | -2.59 | 2.50 | .972 |
| Fear in Posts | -0.11 | 0.13 | -0.36 | 0.13 | .367 |
| Sadness in Posts | -0.01 | 0.08 | -0.17 | 0.14 | .870 |

Table S20. Linear regression model of information sources on tentative tone in the comments.

| Tentative | Estimate | SE | Lower 95% CI | Upper 95% CI | p |
| --- | --- | --- | --- | --- | --- |
| Intercept | 0.21 | 0.07 | 0.08 | 0.34 | .002** |
| Health Media | -0.09 | 0.12 | -0.32 | 0.15 | .474 |
| Hospital | -0.09 | 0.06 | -0.21 | 0.04 | .175 |
| Third-party Fact Checker | -0.08 | 0.14 | -0.36 | 0.20 | .560 |
| Post Word Count | <.001 | <.001 | <.001 | <.001 | .041* |
| Comment Word Count | <.001 | <.001 | <.001 | 0.001 | .002** |
| Page Follower | <.001 | <.001 | <.001 | <.001 | .457 |
| Tentative in Posts | 0.16 | 0.07 | 0.03 | 0.30 | .020* |
| Confident in Posts | 0.21 | 0.07 | 0.07 | 0.34 | .003** |
| Analytical in Posts | -0.01 | 0.06 | -0.12 | 0.11 | .911 |

Table S21. Linear regression model of information sources on confident tone in the comments.

| Confident | Estimate | SE | Lower 95% CI | Upper 95% CI | p |
| --- | --- | --- | --- | --- | --- |
| Intercept | 0.02 | 0.07 | -0.11 | 0.16 | .720 |
| Health Media | 0.03 | 0.12 | -0.20 | 0.27 | .776 |
| Hospital | 0.09 | 0.06 | -0.03 | 0.21 | .155 |
| Third-party Fact Checker | 0.15 | 0.14 | -0.12 | 0.43 | .280 |
| Post Word Count | <.001 | <.001 | <.001 | 0.001 | .004** |
| Comment Word Count | <.001 | <.001 | <.001 | <.001 | .856 |
| Page Follower | <.001 | <.001 | <.001 | <.001 | .774 |
| Tentative in Posts | 0.03 | 0.07 | -0.11 | 0.17 | .674 |
| Confident in Posts | 0.00 | 0.07 | -0.14 | 0.14 | .973 |
| Analytical in Posts | 0.01 | 0.06 | -0.10 | 0.12 | .868 |

Table S22. Linear regression model of information sources on analytical tone in the comments.

| Analytical | Estimate | SE | Lower 95% CI | Upper 95% CI | p |
| --- | --- | --- | --- | --- | --- |
| Intercept | 0.14 | 0.07 | 0.00 | 0.28 | .057. |
| Health Media | 0.15 | 0.13 | -0.10 | 0.40 | .238 |
| Hospital | -0.01 | 0.07 | -0.14 | 0.13 | .936 |
| Third-party Fact Checker | 0.17 | 0.15 | -0.13 | 0.47 | .274 |
| Post Word Count | <.001 | <.001 | <.001 | <.001 | .223 |
| Comment Word Count | <.001 | <.001 | <.001 | 0.001 | .017* |
| Page Follower | <.001 | <.001 | <.001 | <.001 | .573 |
| Tentative in Posts | 0.19 | 0.08 | 0.04 | 0.34 | .015* |
| Confident in Posts | 0.06 | 0.08 | -0.09 | 0.21 | .425 |
| Analytical in Posts | 0.15 | 0.06 | 0.03 | 0.27 | .016* |
